# Kidney Autotransplantation and Orthotopic Kidney Transplantation: Two Different Approaches for Complex Cases

**DOI:** 10.1155/2022/9299397

**Published:** 2022-08-03

**Authors:** Alberto Artiles Medina, Victoria Gómez Dos Santos, Víctor Díez Nicolás, Vital Hevia Palacios, Mercedes Ruiz Hernández, Inés Laso García, Marina Mata Alcaraz, Cristina Galeano Álvarez, Miguel Ángel Jiménez Cidre, Fernando Arias Fúnez, Milagros Fernández Lucas, Francisco Javier Burgos Revilla

**Affiliations:** ^1^Department of Urology, Hospital Universitario Ramón y Cajal, Instituto Ramón y Cajal de Investigación Sanitaria (IRYCIS), University of Alcalá, Madrid, Spain; ^2^Department of Nephrology, Hospital Universitario Ramón y Cajal, Instituto Ramón y Cajal de Investigación Sanitaria (IRYCIS), Madrid, Spain

## Abstract

**Introduction:**

Transplantation surgery teams often have to face complex cases. In certain circumstances, such as occlusion of the iliac vessels or prior pelvic surgery, heterotopic kidney transplantation may not be feasible and orthotopic kidney transplantation (OKT) could be a good alternative. Kidney autotransplantation (KAT) has been described as a potential treatment for complex renovascular, ureteral, or neoplastic conditions. There are scarce data regarding the complications and outcomes of these procedures; therefore, we present our experience.

**Materials and Methods:**

We retrospectively analysed the medical records of both 21 patients who had received OKT and 19 patients who underwent KAT between 1993 and 2020. We collected demographic features and data regarding surgical technique, complications, and graft outcomes. Kidney graft survival was calculated using Kaplan–Meier survival analysis.

**Results:**

Regarding OKT, in 15 (71.43%) cases, it was the first kidney transplantation. The most common indication was the unsuitable iliac region due to vascular abnormalities (57.14%). The early postoperative complication rate was high (66.67%), with 23.81% of Clavien grade 3b complications. During the follow-up period (mean 5.76 -SD 6.15- years), we detected 9 (42.85%) graft losses. At 1 year, the survival rate was 84.9%. Concerning KAT, the most frequent indication was ureteral pathology (52.63%), followed by vascular lesions (42.11%). The overall early complication rate was 42.11%. During the follow-up period (mean of 4.47 years), 4 (15.79%) graft losses were reported.

**Conclusions:**

Although OKT and KAT have high complication rates, these techniques can be considered as two valuable approaches for complex cases, in the absence of other therapeutic options.

## 1. Introduction

Renal transplantation is the treatment of choice for patients with end-stage renal disease (ESRD) for increasing life expectancy and for improving the quality of life. The increasing age of recipients, along with the presence of comorbidities, has increased the transplantation's complexity from both the surgical and medical points of view over the last decades. Furthermore, many complex situations cannot be solved using the conventional heterotopic kidney transplantation (HKT). Orthotopic renal transplantation (OKT) is a technically challenging but valid alternative for patients who are unsuitable candidates for HKT [1]. Nowadays, a significant part of patients admitted to the waiting list have previous transplants or severe vascular atheromatosis and are not candidates for HKT for these reasons. In these cases, OKT could be an alternative [2].

**Figure 1:**
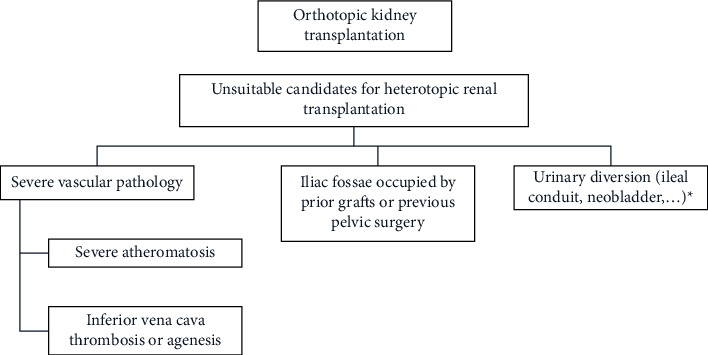
Indications for orthotopic kidney transplantation (OKT). ^*∗*^OKT was used in kidney transplant recipients with a prior urinary diversion in our initial experience.

**Figure 2:**
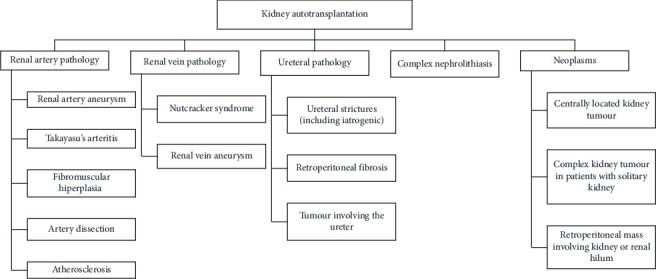
Indications for kidney autotransplantation (KAT).

Kidney autotransplantation is an established but rarely used therapy in cases of renal vessel lesions, tumours of the kidney and ureter, long-distance ureter lesions, complex nephrolithiasis, and retroperitoneal fibrosis [3], which could allow to preserve partly or completely the renal unit and to avoid the risk of chronic kidney disease (CKD) and the potential need for allogeneic transplantation and its associated complications. Figures 1 and 2 summarize the indications of these two techniques used in complex cases.

### 1.1. OKT Technique

The surgical technique of OKT was first described by Gil-Vernet et al. in 1978, and it consists of a retroperitoneal approach to the splenic hilum via lumbotomy and was initially developed to treat hypertension secondary to left renal artery stenosis. After a nephrectomy is performed through a left lumbotomy, the vein is ligated near the renal parenchyma including its bifurcation. Artery revascularization is obtained with end-to-end anastomosis between the graft renal artery and the native splenic artery (or renal artery or inferior mesenteric artery). Vein revascularization is obtained with end-to-end anastomosis between the graft renal vein and the native renal vein or splenic vein. Finally, the urinary system is reconstructed using pyelo-pyelic anastomosis in most cases [4].

### 1.2. KAT Technique

KAT shares similarities with the traditional surgical techniques for living donor nephrectomy and transplantation. Nephrectomy can be performed through lumbotomy and extraperitoneal access to the lumbar fossa, or using the laparoscopic approach. The renal vessels are carefully dissected and ligated. Gibson incision is performed in the iliac fossa, dissecting the iliac vessels and preparing them for anastomosis. It could be performed before sectioning kidney vessels and ureter, in an attempt to minimize ischemia time. Once retrieved, the kidney is perfused with preservation fluid (e.g., Celsior® at 4°C). Vascular anastomoses to reperfuse the kidney are performed end-to-side to the external iliac vessels according to kidney transplant technique, with nonabsorbable 6–0 sutures. Finally, the reconstruction of the urinary tract is performed depending on each case; when possible (only vascular pathology), ureteral reimplantation is avoided to preserve the natural draining of the bladder trigone. When it is not possible, a stented tunneled extravesical ureteroneocystostomy or direct reimplantation can be chosen [5].

There are scarce data regarding the outcomes of patients who undergo OKT and KAT. According to the literature review performed by Alameddine et al. [6] in 2018 and Musquera et al. [7] in 2020, in relation to KAT and OKT, respectively, in this article our series represents one of the largest series in the literature.

This study aimed to analyse patients' characteristics, indications, complications, and outcomes of OKT and KAT in one of the largest series of these two techniques.

## 2. Materials and Methods

We retrospectively analysed the medical records of patients who underwent OKT or KAT between 1993 and 2020 at our institution. We collected demographic characteristics of the recipients and donors and data regarding surgical technique and complications (early: <30 days or late: ≥30 days after surgery) as well as graft outcomes, including graft function at discharge, 1 month, 1 year, and 5 years, graft survival, and graft loss.

Reviewed demographic data included age, gender, and age-adjusted Charlson comorbidity index (ACCI). Autotransplant outcome was evaluated using graft loss rates and laboratory data (serum creatinine at 1 month, 1 year, and 5 years), because missing data regarding the date of graft loss meant that a survival analysis of KAT was not appropriate.

### 2.1. Definitions

Patients who did not require dialysis following transplantation were defined as having immediate graft function (IGF). Primary nonfunction (PNF) was defined as a permanent loss of allograft function starting immediately after transplantation, without vascular cause. Graft loss results in return to dialysis, retransplantation, or death.

Graft rejection was histologically confirmed (presence of tubulointerstitial inflammation and fibrosis) or clinically suspected. Delayed graft function (DGF) was defined as the need for dialysis in the first posttransplantation week. The main cause of DGF was acute tubular necrosis (ATN).

Non-death-censored graft survival was calculated from the date of transplantation to the date of irreversible graft failure (return to dialysis or retransplantation) or the date of the last follow-up or to the date of death (death with functioning graft is considered as graft loss).

### 2.2. Statistical Analysis

Data were analysed with SPSS software (Statistical Package for Social Sciences for Windows, version 23.0, SPSS Inc., Chicago, IL, USA). Descriptive statistics are shown as mean and standard deviation, or frequency and percentage. Kidney graft survival was calculated using Kaplan–Meier survival analysis.

## 3. Results

### 3.1. OKT

21 patients had received OKT between 1993 and 2020. The primary source of renal grafts was brain death donors. The mean age of the donors was 69.2 (SD 14.53) years. A preference for the selection of left kidneys was observed (72%).  Table 1 contains the donor's main characteristics.

Table 2 shows the baseline features of patients who underwent OKT. Out of 21, in 15 (71.43%) cases it was the first kidney transplantation. The most common indication of OKT (Table 3) was the unsuitable iliac region due to vascular abnormalities, followed by the presence of a prior urinary diversion (especially in the first 10 years of our experience).

Regarding postoperative complications (see Table 4), the early postoperative complication rate was high (66.67%), with 23.81% of Clavien grade 3b complications. We observed 3 vascular complications in the immediate postoperative period: 2 arterial thrombosis that required transplantectomy and one hypoperfusion that required transplantectomy in the same surgical act.

Table 5 represents the summary of graft outcomes. 61.90% of the patients had IGF. During the follow-up period (mean 5.76 (SD 6.15) years), we detected 9 (42.85%) graft losses.

**Figure 3:**
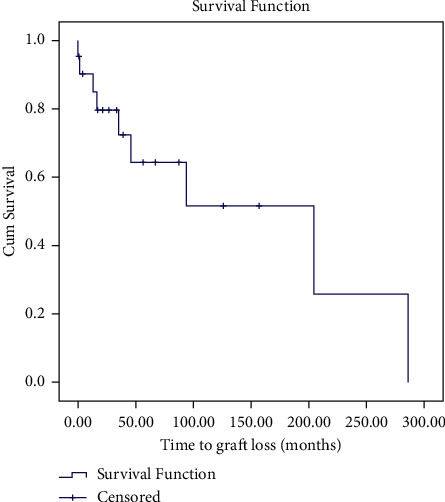
Kaplan–Meier survival curve showing non-death-censored graft survival of OKT grafts.

Kaplan–Meier survival curve showing graft survival is seen in Figure 3. The mean survival was 204.42 (CI 95% 45.47–363.36) months. At 12 months, the survival rate was 84.9%.

### 3.2. KAT

19 patients underwent KAT between 1993 and 2020 at our institution. Patients' demographic characteristics are shown in Table 6. The majority was in the fourth decade of life (mean age 48.33 (SD 17.36) years) and only 3 (15.78%) patients had a history of solitary kidney. Regarding the subgroup of patients with a solitary kidney who underwent KAT, the mean preoperative creatinine and eGFR were 0.87 mg/dL and 66.98 mL/min/1.73 m^2^, respectively. Two patients experienced an improvement in eGFR (change in eGFR of 9.89 and 9.72). In contrast, one patient suffered a decline in renal function during follow-up (a reduction of eGFR in 25.77).

The most frequent indication for KAT was ureteral pathology (52.63%), followed by vascular lesion (42.11%), which was the predominant one in the first years of our series. One rare case of a retroperitoneal residual germ cell tumour was performed in 2020 (Table 7). The case included in our series refers to a patient who had a retroperitoneal mass measured 10 cm and located in the left paraaortic area, after receiving 4 cycles of chemotherapy with adjuvant BEP (bleomycin, etoposide, and cisplatin) in spite of normalized tumour markers.

The overall early complication rate of KAT was 42.11% (Table 8). The rate of Clavien grade ≥3 complications was 5.26%.

Graft outcomes are summarized in Table 9. 3 (15.79%) acute tubular necrosis (ATN) cases were observed in the immediate postoperative period. Additionally, 4 (15.79%) graft losses were reported during the follow-up period (mean of 4.47 years).

## 4. Discussion

Few articles have addressed the issue of OKT and KAT, because these procedures are used to handle high-complexity cases. As a consequence, our knowledge is based on a small number of case series reported by high-volume centers. In light of this lack of data, we report our experience to contribute to widen our knowledge of these techniques and their outcomes.

### 4.1. Indications

Certain conditions of candidates for kidney transplantation make this technique a complex and challenging surgery. For example, atherosclerosis, which is common in ESRD patients on dialysis, can be considered a relative contraindication to HKT in some cases. OKT is a good alternative when the heterotopic technique is not feasible and may play a role in selected patients with aortoiliac unworkable segments, occupied iliac fossae, or even in patients with special urinary tract conditions [8].  Table 10 includes the largest series of OKT, summarizing indications, complication rate, and graft survival.

**Figure 4:**
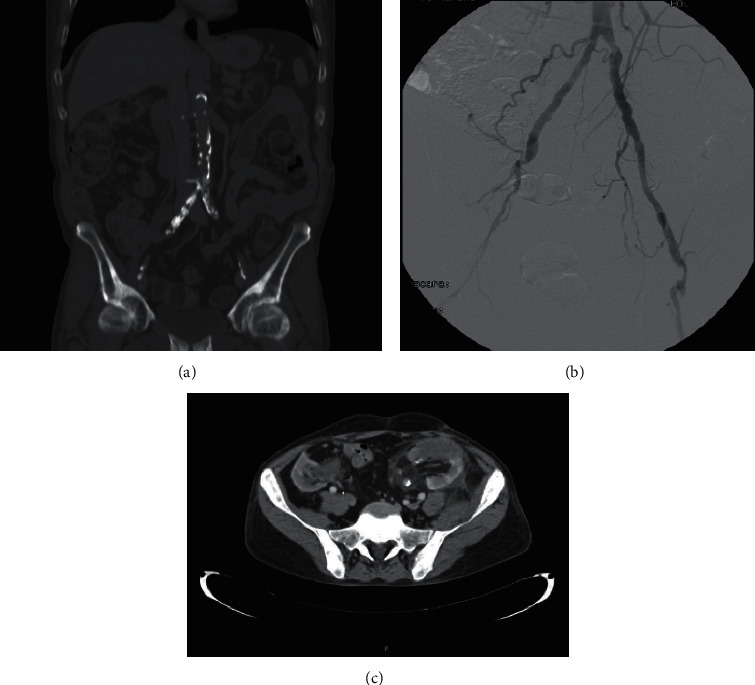
(a). Important aortoiliac calcification. (b). Arteriography showing left external iliac artery stenosis. (c). Bilateral occupation of the iliac fossae by prior kidney grafts.

Prior works have proved that the most common indication of OKT is the unsuitable iliac region mainly due to vascular abnormalities (Figure 4). Our results are in line with these prior findings; in our series, in 57.14% of patients, the OKT was indicated because of vascular reasons.

Kidney transplantation with arterial anastomosis on vascular prosthesis, in selected patients, can offer an alternative to dialysis [13]. According to a survey among 161 transplant surgeons regarding screening and management of patients with aortoiliac occlusive disease (AIOD) for kidney transplantation, there is no uniformity. For example, screening for AIOD is commonly (38.5%) restricted to high-risk patients, and pretransplant vascular procedures to facilitate transplantation are infrequently performed (71.4% mentioned <10 per year) [14].

**Figure 5:**
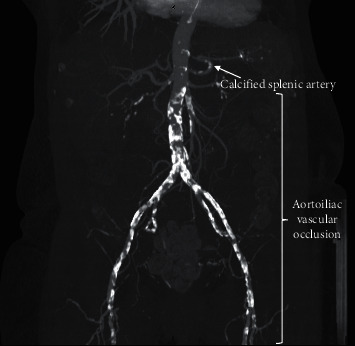
A CT reconstruction showing aortoiliac vascular occlusion and calcified splenic artery. This is a complex case that could require the anastomoses to the native renal vessels.

**Figure 6:**
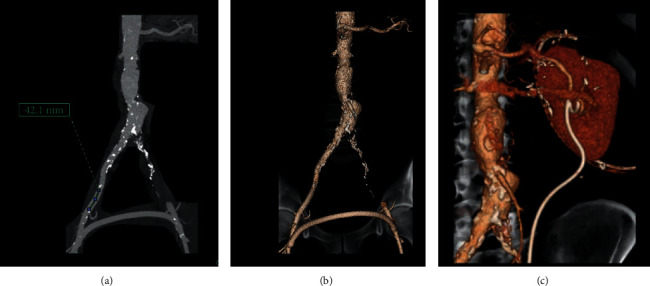
(a and b) represent the CT angiography (reconstruction) in a challenging case of a patient with a left external and common iliac artery chronic total occlusion who underwent femorofemoral bypass. In this case, transplantation in the intact right iliac artery segment (43.1 mm) could be associated with vascular steal following heterotopic transplantation. The image C corresponds to the post-OKT CT angiography.

In spite of the fact that acceptable outcomes have been reported on renal transplant on a prosthetic vessel (ARTPV), some reasons could justify the selection of OKT as a valuable option in patients with AIOD. In selected cases, the indication for OKT is clear. For example, unsuccessful or technically unfeasible vascular procedures, or vascular abnormalities (see Figures 5 and 6). It is essential to define selection criteria prior to this kind of surgery and decide when vascular prostheses for iliac artery reconstruction for transplantation are better than an OKT. Multidisciplinary meetings including a vascular surgeon are a key aspect in the management of these medically complex patients for transplantation. In some complex cases of AIOD, patients have asymptomatic disease (without intermittent claudication) and therefore, the indication of vascular prosthesis is controversial. During long-term follow-up, these patients could require interventions (e.g., severe ipsilateral external iliac artery stenosis distal to prosthesis) that could compromise the transplant's survival. Moreover, these patients usually have a high surgical risk and an increased mortality risk because of cardiovascular comorbidity. In many cases, kidney transplantation could be delayed due to medical complications following vascular interventions prior to kidney transplantation and less frequently, eventually, it could be absolutely contraindicated. Regarding renal transplant on a prosthetic vessel, there is a higher rate of complications when prosthetic vessel replacement is combined simultaneously with kidney transplantation. However, previous vascular surgery could be justified to facilitate living donor kidney transplantation or in patients with common blood groups, in whom the estimated waiting time to transplantation is shorter. OKT offers an alternative to the need for vascular reconstructive surgery. In this context, the evaluation of these patients in referral centers and the participation of vascular surgeons in decision-making is highly recommended.

Sagban et al. performed an extensive literature research and contrasted the results with their experience on ARTPV. 170 ARTPV patients reported in 18 published articles were analysed. The literature analysis revealed that a prosthetic bypass for revascularization prior to transplantation is the preferred approach (58% of published cases). Regarding complications, redo surgery was necessary in 15% of cases, 30-day mortality was on average 6%, and most deaths occurred in cases of combined kidney transplantations and simultaneous ARTPV. Furthermore, the 5-year graft survival rate was reported in 12 studies and ranged from 17% to 100% [15].

The data from our series were initially reported by Hevia et al. [2]. They published an article reporting complications and outcomes of a series of 9 patients. These cases are included in the present series. The most common indication for OKT was an unsuitable iliac region in six (66.6%) and abnormalities in the low urinary tract or urinary diversion in three (33.3%). The latter is a common indication in the first period of our series and refers to patients with a history of radical cystectomy and urinary diversion (such as ileal conduit or neobladder) due to bladder cancer, with or without unsuitable pelvic vessels for transplantation. In this context, the indication for OKT is to avoid the dissection of the iliac vessels, which can be challenging after lymphadenectomy [7]. Similarly, in the largest series by Musquera et al. [4] (Table 10), severe aortoiliac atherosclerosis (41.7%) was the most common reason to perform an OKT, although in this study it was followed by bilaterally retained iliac fossae (28.9%).

Retransplantation rates have drastically increased to almost 25% over the past decades. Schachtner et al. studied 111 patients who underwent their second kidney transplantation and compared the outcomes between prior removed, or retained, allografts. Their data suggest higher cellular presensitization among kidney transplantations with the previous allograft removed, and it is associated with higher rates of acute cellular rejection and lower graft survival [16]. In addition, retained bilateral iliac fossa kidney transplant could make technically difficult or unfeasible heterotopic kidney retransplantation, particularly in prior transplant recipients in whom both iliac vessels have been previously accessed. In our series, an unsuitable iliac region due to prior transplantations (19.05%) was the third most common indication.

Other conditions, like inferior vena cava (IVC) thrombosis, agenesia, or stenosis, could require OKT. Chan et al. have published a series of 3 cases of OKT in this scenario [12].

**Figure 7:**
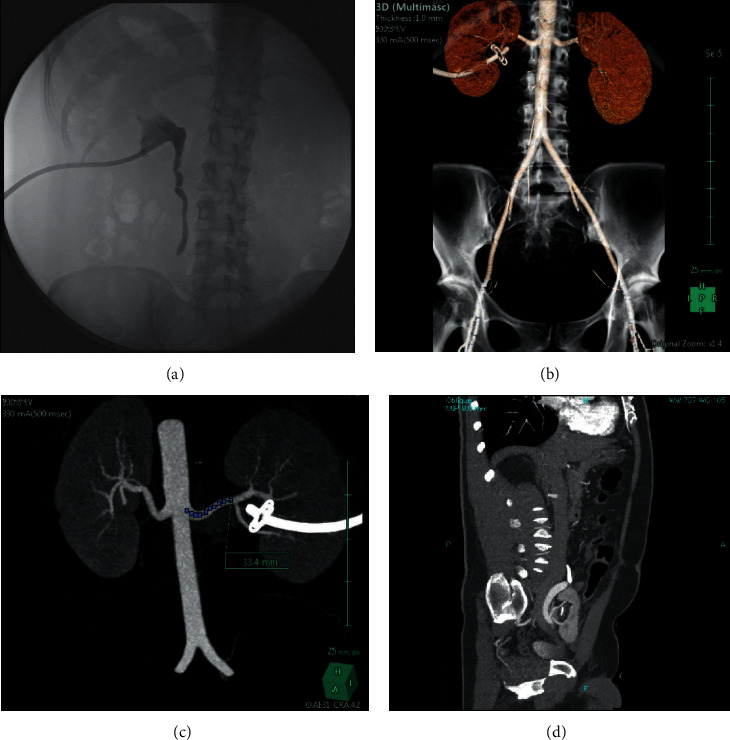
(a). Antegrade pyelography showing ureteral stop secondary to an iatrogenic ureteral lesion during open surgery. (b) and (c). Three-dimensional computed tomography reconstruction of the artery Ao, abdominal aorta. (d) Sagittal CT image showing KAT location and vasculature.

On the other hand, KAT is an attractive approach because it addresses the underlying pathology while eliminating the problems associated with allogeneic transplantation [6]. There are limited data regarding the outcomes of patients who underwent this procedure (Table 11). Moghadamyeghaneh et al. investigated the outcomes of such patients using a nationwide inpatient sample database (2002 to 2012). A total of 817 patients underwent kidney autotransplantation. The most common indication of surgery was renal artery pathology (22.7%) followed by ureter pathology (17%) [21]. In contrast, in our experience, the most prevalent indication of KAT was ureteral pathology (52.63%) (Figure 7). Vascular lesion was the most frequent indication in the first period of our experience. Some series have specifically included vascular causes for KAT; Chiche et al. [17] reported their experience treating fibromuscular dysplasia using KAT technique. Similarly, Duprey et al. performed KAT in order to surgically remove renal artery branch aneurysms. In contrast, other series share the ureteral pathology as the most frequent indication. For example, Tran et al. [18] reported that 78.8% of cases of KAT are due to ureteral lesions.

Duprey et al. [20] evaluated the long-term outcomes of renal revascularization by ex vivo renal artery reconstruction and autotransplantation for renal artery branch aneurysms in 65 patients and presented favourable results. Over the past decades, advances in this field have contributed to reduce the number of indications of KAT for vascular reasons. Nowadays, technical improvements in interventional endovascular treatment have led to a more widespread use of endoluminal renal artery revascularization and extension of the indications for this type of therapy [22], instead of KAT in many renal artery pathologies.

Pursuing the principle of nephron-sparing technique, KAT may allow in some cases the excision of renal masses in patients with a solitary kidney, or in those with large-sized masses, centro-renal location, and when the in vivo partial nephrectomy is technically difficult. In our series, only one KAT was performed due to oncological reasons, particularly a residual retroperitoneal mass in a patient with a history of germ cell cancer [23].

In conclusion, aortoiliac occlusive disease and ureteral lesions are the leading indications for OKT and KAT, respectively.

### 4.2. Complications

Musquera et al. reported the results of OKT in their center and compared indications, surgical techniques, and long-term results from two different periods (before and after February 1987). They noted that the overall graft and patient survival were similar between orthotopic and heterotopic kidney transplants performed during the same period. These authors concluded that OKT is a good alternative with acceptable rates of urologic and vascular complications for those patients for whom heterotopic transplant is considered unsuitable [4].

As previously reported, the vascular complication rate of OKT ranges from 2.8 to 9.6%. In our series, 3 of 21 (14.28%) patients developed vascular complications (two arterial thrombosis and one hypoperfusion that required transplantectomy). According to Musquera et al., complications included renal artery stenosis in 3.6%, renal artery thrombosis in 4.8%, and renal vein thrombosis in 1% [24]. Urinary fistula occurred in 9.5% [4]. We only identified one urinary leak, and there were no clinically significant arterial stenoses in our series.

The initial experience in elective OKT of Gil-Vernet et al. [9] informed us of a vascular complication rate of 2.8%. That rate has increased (9.6%) in the updated series, due to the inclusion of medically complex patients and the growing use of grafts from marginal kidney donors.

**Figure 8:**
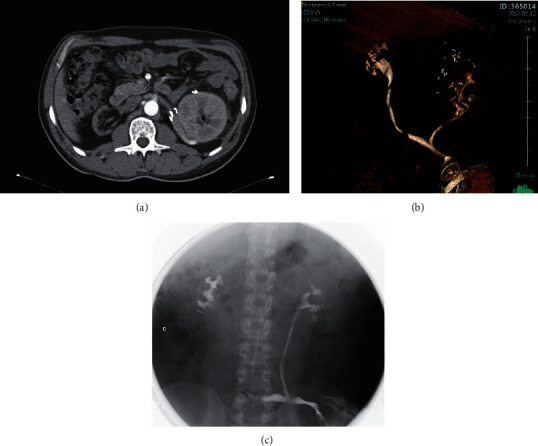
(a). CT scan showing an orthotopic kidney transplantation with the venous reconstruction of the left renal vein. (b) (adapted from Hevia et al.) and (c) are a reconstructed CT image of the urinary system and intravenous urography, respectively, representing a ureteroureterostomy in orthotopic renal transplantation in a patient with a history of myelomeningocele and cystectomy with cutaneous ureterostomy.

Since the update on OKT published in 2010 by Musquera, no more large series have been reported. Some small series or case reports are being described in the literature. The orthotopic position has shown good recipient and graft results with an acceptable complication rate in selected patients [7]. The overall complication rate is variable among previous studies (33.3–100%). In our case series, the complication rate within 30 days after surgery was 66.67%. It is higher than previously reported data from Hevia et al. [2] (33.3%).  Figure 8 illustrates the venous and urinary anastomoses in an OKT.

Regarding KAT outcomes, the initial experience of our series was published by Ruiz et al., including 15 patients. We reported a complication rate of 46.7%. In our updated series, the overall early complication rate was 42.11%. This rate is similar to Moghadamyeghaneh et al. (46.2%) who have published the largest series [6]. However, other authors have reported lower overall complication rates, such as Chiche et al. [17] (15.79%) or Cowan et al. [19] (12.9%). Additionally, recently, robot-assisted KAT has been proved to be a safe approach [25].

To sum up, OKT and KAT are highly specialized techniques in the field of kidney transplantation, with high complication rates.

Furthermore, some groups have reported their series of robot-assisted kidney autotransplantation (RAKAT) and indicated that this technique is safe and feasible in selected patients with complex ureteral or renal pathology [25]. This approach has been further used to robotically transplant kidneys from living donors.

### 4.3. Transplantation Outcomes

Regarding OKT, the initial analysis of our series (Hevia et al.) revealed a DGF rate of 22.2% (2/9) [2]. In our updated series, we found DGF in 8 of 21 patients (38.09%). As previously reported in the literature, the graft survival of OKT is comparable to conventional heterotopic kidney transplantation. In the large series study by Musquera et al., the long-term graft survival was comparable between OKT and heterotopic kidney transplant (34.5% vs 29.2%, respectively, at 20 years) [24]. Our work has revealed that at 12 months, the survival rate was 84.9%. These data are similar to those reported by Musquera et al. and Hevia et al. [2] According to the previously reported series by Paduch et al. [10] and Rodrigues et al. [11], graft rates of 100% during follow-up have been informed.

According to Ruiz et al., after a mean follow-up of 73.1 months (range 7–312), 80% of the patients who underwent KAT have a functioning graft [5]. Our current analysis showed that 15 of 19 patients (78.94%) had a functioning graft after a mean follow-up of 4.47 years. In the series of Moghadamyeghaneh et al., a kidney transplant failure rate was observed in 10.7% of patients [21]. It accounts for 15.79% of patients in our series. Other series have also reported graft loss rates ranging from 10 to 15%, such as Chiche et al. [17] (14%), Tran et al. [18] (9.6%), and Duprey et al. (12.3%).

The present findings and the review of the published series help to become established OKT and KAT as two valuable approaches for complex cases.

## 5. Conclusions

Given the rising number of recipients suffering from vascular pathology, OKT is becoming a growing technique that lets us to avoid other techniques such as the use of arterial prostheses. However, this is a controverted issue. On the other hand, regarding KAT, its indications have remained solid and stable over the last years and are mainly ureteral iatrogenic lesions.

Transplantation surgery teams increasingly have to face complex scenarios. In these challenging cases, although OKT and KAT have high complication rates, these techniques can be useful approaches to consider, with promising graft outcomes, in the lack of another surgical option.

## Figures and Tables

**Table 1:** Donors' characteristics.

Variable	Value
Donor age in years, mean (SD)	69.2 (14.53)
Donor terminal serum creatinine, mean (SD)	0.77 (0.23)
Proportion of glomerulosclerosis, mean (SD)	7.5 (4.89)
Cold ischemia time, hours, mean (SD)	18.59 (2.21)

**Table 2:** Baseline characteristics.

Variable	Value
Age in years, mean (SD)	52.66 (13.99)
Female vs male, *n* (%)	5 (23.81%) vs 16 (76.19%)
aCCI, mean (SD)	4.47 (1.96)

Number of prior transplants	
0	15 (71.43%)
1	2 (9.52%)
2	2 (9.52%)
3	1 (4.76%)
5	1 (4.76%)

ESRD causes	
Diabetic nephropathy	4 (19.05%)
Interstitial nephritis	4 (19.05%)
Unknown origin	3 (14.29%)
Glomerulopathies	5 (23.81%)
Nephroangiosclerosis	1 (4.76%)
Cyclosporine nephropathy	1 (4.76%)
Kidney dysplasia	1 (4.76%)
Others (secondary to obstructive uropathy or reflux nephropathy)	2 (9.52%)

**Table 3:** Indications of OKTs and surgical data.

Variable	Value
Indication	
Unsuitable iliac region due to vascular abnormality	12 (57.14%)
Unsuitable iliac region due to prior transplantations	4 (19.05%)
*Prior urinary diversion*	5 (23.81%)
Ileal conduit	4
Cutaneous ureterostomy	1
Arterial anastomosis	
Donor renal artery to the native splenic artery	20 (95.24%)
Donor renal artery to the aorta	1 (4.76%)
Venous anastomosis	
Donor renal vein to the native renal vein	18 (85.71%)
Donor renal vein to the native splenic vein	2 (9.52%)
Donor renal vein to the inferior cava vein	1 (4.76%)
Urinary reconstruction	
Ureteroureterostomy	14 (66.67%)
Pyelopyelostomy	6 (28.57%)
Pyeloureterostomy	0
Ureteric implantation in ileal conduit	1 (4.71%)
Operation time, min, mean (SD)	283.46 (70.22)

**Table 4:** Early and late postoperative complications of OKT.

Variable	Value
Early postoperative complications (≤30 days)	14 (66.67%)

*Surgical complications*	
(i) Arterial thrombosis that required transplantectomy, *n* (%)	2
(ii) Hypoperfusion that required transplantectomy, *n* (%)	1
(iii) Bleeding, *n* (%)	1
(iv) Acute urinary retention after urinary catheter removal, *n* (%)	2
(v) Perigraft fluid collection, *n* (%)	1
(vi) Urinary leak, *n* (%)	1
(vii) Surgical site infection, *n* (%)	1

*Medical complications*	
(i) Decompensated heart failure, *n* (%)	1
(ii) Gastrointestinal bleeding, *n* (%)	1
(iii) Febrile urinary tract infection, *n* (%)	1
(iv) Respiratory septic shock (ventilator-associated pneumonia), *n* (%)	1
(v) acute rejection, *n* (%)	1

Early postoperative complications (≤30 days) according to Clavien-Dindo classification	
2	6 (28.57%)
3a	2 (9.52%)
3b	5 (23.81%)
5	1 (4.76%)

Hospital stay, mean (SD)	14.36 (7.08)
Time to double *J* removal, days, mean (SD)	25.85 (14.68)
Late postoperative complications (>30 days)	6 (28.57%)

*Surgical complications*	
(i) Obstructive lymphocele, *n* (%)	1
(ii) Pancreatic fistula, *n* (%)	1

*Medical complications*	
(i) Septic shock secondary to pyelonephritis, *n* (%)	1
(ii) Clostridium difficile associated diarrhoea, *n* (%)	1
(iii) Urinary tract infection, *n* (%)	1
(iv) Graft rejection, *n* (%)	1

Late postoperative complications (>30 days) according to Clavien-Dindo classification	
2	3 (13.21%)
3a	1 (4.76%)
3b	1 (4.76%)
5	1 (4.76%)

**Table 5:** OKT outcomes.

Variable	Value
Mean follow-up in years	5.76 (6.15)
Immediate graft function, *n* (%)	13 (61.90%)
DGF, *n* (%)	8 (38.09%)
PNF, *n* (%)	0
^*∗*^Lost graft function due to vascular reasons	3 (14.28%)

*At discharge*	
Creatinine level, mean (SD)	2.02 (0.91)
eGFR at discharge, mean (SD)	45.28 (24.17)

*At 1 month after surgery*	
Creatinine level, mean (SD)	1.79 (0.66)
eGFR, mean (SD)	43.95 (17.75)

*At 1 year after surgery*	
Creatinine level, mean (SD)	2.41 (3.01)
eGFR at 1 year after surgery, mean (SD)	47.53 (SD 27.04)

*At 5 years after surgery*	
Creatinine level, mean (SD)	1.47 (SD 0.24)
eGFR, mean (SD)	46.28 (SD 6.83)

Overall mortality, *n* (%)	7 (33.33%)

*Cause of death*	
Gastrointestinal bleeding, *n* (%)	1
Septic shock, *n* (%)	2
Cardiac arrest, *n* (%)	1
Melanoma, *n* (%)	1
Intestinal obstruction, *n* (%)	1
Acute arterial limb ischemia, *n* (%)	1

Functioning graft, *n* (%)	12 (66.67%)
Graft loss, *n* (%)	9 (42.85%)
Graft rejection, *n* (%)	3 (14.29%)

**Table 6:** Baseline characteristics of the KAT series.

Variable	Value
Age in years, mean (SD)^*∗*^	48.33 (17.36)
aCCI, mean (SD)	2.44 (2.60)
Preoperative creatinine, mean (SD)	1.04 (SD 0.38)
Preoperative eGFR, mean (SD)	78.96 (SD 36.88)
Solitary kidney, *n* (%)	3 (15.78%)

^*∗*^excluded pediatric patients.

**Table 7:** Indications of KATs and surgical data.

Variable	Value
Indication	
**Ureteral stricture, n (%)**	10 (52.63%)
Following open surgery	4
Following ureteroscopy	4
Secondary to retroperitoneal fibrosis	1
Secondary to Crohn's disease	1
**Vascular lesion, n (%)**	8 (42.11%)
Renal artery stenosis, *n* (%)	4
Renal artery fibromuscular dysplasia, *n* (%)	2
Takayasu disease, *n* (%)	1
Renal artery aneurysm, *n* (%)	1
**Retroperitoneal mass, n (%)**	1 (5.26%)

Nephrectomy approach, *n* (%)	
Open	15 (78.94%)
Laparoscopic	4 (21.05%)
Operation time, min, mean (SD)	390 (165.87)
Ureteral disinsertion	13 (68.42%)

*Type of ureteral reconstruction*	
Ureteroneocystostomy	
(i) Direct reimplantation, *n* (%)	2 (15.38%)
(ii) Extravesical technique, *n* (%)	10 (76.92%)
Ureteroureterostomy, *n* (%)	1 (7.69%)
Cold ischemia time, min, mean (SD)	84.28 (67.84)

**Table 8:** Early and late postoperative complications of KAT.

Variable	Value
Early postoperative complications (≤30 days)	8 (42.11%)

*Surgical complications*	
Surgical site infection	
Superficial	2
Deep	1
Thrombosis of renal vein, *n* (%)	1
Arterial thrombosis, *n* (%)	1

*Medical complications*	
Acute tubular necrosis, *n* (%)	1
Decompensated heart failure, *n* (%)	1
Fever of unknown origin, *n* (%)	1

Hospital stay, days, mean (SD)	10.44 (5.38)

Early postoperative complications (≤30 days) according to Clavien-Dindo classification	
1	5 (26.32%)
2	2 (10.53%)
3b	1 (5.26%)

Late postoperative complications (>30 days)	
Obstructive uropathy, *n* (%)	1 (5.26%)
Late postoperative complications (>30 days) according to Clavien-Dindo classification	1
3a	1 (5.26%)

**Table 9:** KAT outcomes.

Variable	Value
Mean follow-up in years	4.47
Acute tubular necrosis in the immediate postoperative period, *n* (%)	3 (15.79%)

*At 1 month after surgery*	
Creatinine level, mean (SD)	0.97 (0.47)
eGFR, mean (SD)	91.43 (38.06)

*At 1 year after surgery*	
Creatinine level, mean (SD)	1.18 (0.34)
eGFR, mean (SD)	70.83 (SD 24.49)

*At 5 years after surgery*	
Creatinine level, mean (SD)	1.07 (SD 0.24)
eGFR, mean (SD)	76.94 (SD 20.82)

Creatinine level at 1 year after surgery in patients with solitary kidney (*n* = 3)	1.25 (SD 0.41)
eGFR at 1 year after surgery in patients with solitary kidney (*n* = 3)	64.92 (SD 29.06)

Overall mortality, *n* (%)	0
Functioning graft, *n* (%)	15 (78.94%)
Graft loss during follow-up, *n* (%)	4 (15.79%)

*Early graft loss*	
(i) Arterial thrombosis	1
(ii) Venous thrombosis	1

*Late graft loss*	
(i) Obstructive uropathy	1
(ii) Unknown origin	1
Clinically significant stenosis in the arterial anastomosis, *n* (%)	0

**Table 10:** Largest series of OKT published in the literature to date.

Author	Year	Sample	Indications	Complication rate	Graft survival
Gil-Vernet et al. [9]	1989	139	Alternative to HKT (elective surgery)	Vascular: 4/139 (2.8%)	Graft loss rate: 7/139 (5%)
				Urinary: 5/139 (2.8%)	

Paduch et al. [10]	2001	5	Severe iliac atherosclerosis: 3	NA	Graft survival: 100% during follow-up (from 6 months to 5 years)
			Retained bilateral iliac fossa kidney transplant: 2		

Rodrigues et al. [11]	2004	4	Bilateral occlusion of common iliac arteries: 4	NA	Graft survival: 100% (9 days–14 months)

Musquera et al. [4]	2010	223 (^*∗*^84 in the modern period)	Severe iliac atherosclerosis: 41.7%	Vascular: 13/84 (9.6%)	Graft survival at 1 year: 86.4%
			Bilaterally retained iliac fossae from a previous kidney transplant: 28.9%	Urinary: 16/84 (19.1%)	
			Elective: 4.5%		

Hevia et al. [2]	2014	9	Unsuitable iliac region: 67%	Early: 33.3% (3/9)	Graft survival at 1 year: 88.9%
			LUT abnormalities/urinary diversion: 33%	Late: 22.2% (2/9)	

Chan et al. [12]	2019	3	IVC thrombosis or stenosis	Early: 100% (2 Clavien grade 2 and 1 Clavien grade 3a)	NA

**Table 11:** Largest published series of KAT.

Author	Year	Sample	Indications	Complication rate	Graft loss
Chiche et al. [17]	2003	57	Vascular pathology: Fibromuscular dysplasia for 34 RAT procedures in 30 patients, Takayasu's disease for 26, and others	9/57 (15.79%)	8/57 (14.04%)

Tran et al. [18]	2015	52	Vascular pathology: 3.8%	4 patients had early complications and 8 had late complications	5/52 (9.61%)
			Ureteral pathology: 78.8%		
			Malignancy: 13.4%		

Cowan et al. [19]	2015	51	Vascular anomalies: 18.5%	Overall: 12.9%	2/51 (3.92%)
			Loin pain hematuria syndrome/chronic kidney pain: 31.5% Ureteral stricture: 20.4%	Grade 3a or greater: 14.8%	

Duprey et al. [20]	2016	65	Renal artery branch aneurysms	12 (18%) major postoperative complications	8/65 (12.3%)

Moghadamyeghaneh et al. [6]	2017	817	Vascular pathology: 22.7%	46.2%	10.7%
			Ureteral pathology: 17%		
			Malignancy: 14.9%		

## Data Availability

All data generated or analysed during this study are included within the article.
